# A Case of Esophageal Retention Cyst with High Fluorodeoxyglucose Uptake on PET/CT Scan

**DOI:** 10.70352/scrj.cr.25-0348

**Published:** 2025-07-29

**Authors:** Byonggu An, Hiroshi Yamamoto, Yasumitsu Oe, Takeshi Togawa, Kazumi Shimamoto, Hiromitsu Ban, Tetsuya Abe, Yuki Morimoto, Takashi Matsunaga, Toru Imagami, Akira Sogawa, Nobuyuki Takao, Shizuki Takemura, Akiyoshi Mizumoto

**Affiliations:** 1 Department of Digestive Surgery, Omi Medical Center, Kusatsu, Shiga, Japan; 2 Department of Surgery, Japan Community Healthcare Organization Shiga Hospital, Otsu, Shiga, Japan; 3 Department of Gastroenterology, Omi Medical Center, Kusatsu, Shiga, Japan; 4 Department of Gastroenterological Surgery, Aichi Cancer Center Hospital, Nagoya, Aichi, Japan; 5 Department of Diagnostic Pathology, Omi Medical Center, Kusatsu, Shiga, Japan

**Keywords:** esophageal retention cyst, PET/CT, intense FDG uptake

## Abstract

**INTRODUCTION:**

Esophageal retention cysts are rare, benign lesions that can mimic submucosal tumors. Their clinical presentation and imaging characteristics may lead to diagnostic challenges, particularly when fluorodeoxyglucose-positron emission tomography/CT (FDG-PET/CT) shows increased uptake, raising suspicion of malignancy.

**CASE PRESENTATION:**

A 77-year-old man presented with epigastric pain. Upper gastrointestinal endoscopy revealed an esophageal mass, prompting referral to our hospital. Endoscopic ultrasonography (EUS) identified a hypoechoic submucosal tumor with multiple cystic components in the lower esophagus. However, EUS-guided fine-needle aspiration (EUS-FNA) did not yield a definitive diagnosis. CT scan demonstrated a 60-mm space-occupying lesion (SOL) in the lower thoracic esophagus with peripheral contrast enhancement and a central low-density area. MRI revealed a SOL in the lower esophagus with high signal intensity on T2-weighted images and moderate signal intensity on T1-weighted images. The lesion contained cystic components exhibiting high T2 and low T1 signal intensities. FDG-PET/CT revealed intense FDG uptake, increasing from maximum standardized uptake value (SUVmax) 11 to 18 over time. Given the large size of the tumor, symptomatology, and inability to exclude malignancy—particularly high-risk gastrointestinal stromal tumor—surgical resection was performed. Laparoscopic esophagectomy was conducted using intraoperative endoscopy for tumor identification. The esophagus was transected proximally using a linear stapler, followed by extracorporeal gastric conduit reconstruction and the overlap technique was used to perform an esophagogastric anastomosis. Postoperatively, anastomotic leakage was detected on day 3, requiring emergency reoperation. The leak had resolved by POD 26, and the patient was discharged on day 48 after the second surgery (day 51 after the initial surgery). Histopathological examination revealed multiple cysts of varying sizes within the lamina propria, lined by columnar epithelium, with no evidence of malignancy. The final diagnosis was esophageal retention cyst.

**CONCLUSIONS:**

This case highlights the diagnostic challenge of esophageal retention cysts with high FDG uptake. While PET/CT is essential in oncologic imaging, FDG accumulation does not always indicate malignancy.

## Abbreviations

CEA
: carcinoembryonic antigen
EUS
: endoscopic ultrasonography
FDG
: fluorodeoxyglucose
FNA
: fine-needle aspiration
GIST
: gastrointestinal stromal tumor
PET
: positron emission tomography
SOL
: space-occupying lesion

## INTRODUCTION

Esophageal retention cysts are acquired lesions of unknown etiology, characterized by dilation of the submucosal glands.^1)^ They typically appear as polyps or nodular defects in the distal esophagus and may cause signs or symptoms such as dysphagia or chest pain.^2,3)^ Symptomatic cases are often effectively managed with endoscopic mucosal resection rather than surgery.^1)^

PET/CT is widely utilized for the diagnosis and staging of esophageal cancer. While PET/CT has superior sensitivity in detecting esophageal lesions compared to CT alone,^4)^ increased FDG uptake can also occur in benign conditions such as esophagitis and schwannomas, and may mimic malignancy on PET/CT imaging.^5–8)^

Here, we present a rare case of an esophageal retention cyst that exhibited intense FDG uptake on PET/CT and discuss the diagnostic challenges associated with this condition.

## CASE PRESENTATION

**Fig. 1:**
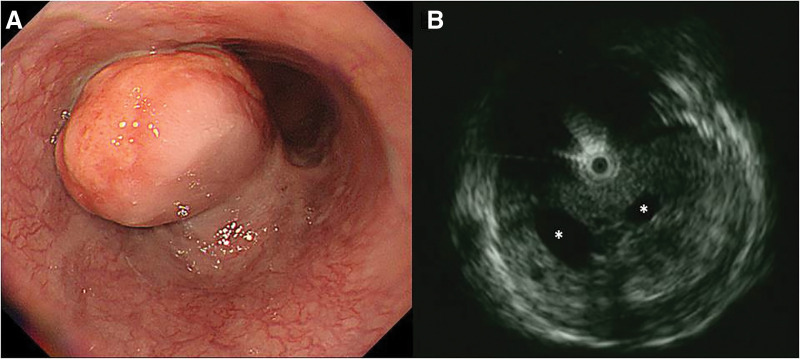
(**A**) An endoscopic examination identified a submucosal tumor in the lower esophagus, approximately 40 cm from the incisors. (**B**) EUS revealed a hypoechoic mass with multiple cystic structures (asterisks). EUS, endoscopic ultrasonography

A 77-year-old man presented to a local clinic with epigastric pain. The results of biochemical testing of peripheral blood samples at the time of presentation were unremarkable, except for a mildly elevated CEA level of 6.6 ng/mL. Upper gastrointestinal endoscopy revealed an esophageal mass, prompting his referral to our hospital. A subsequent endoscopic examination identified a submucosal tumor in the lower esophagus, approximately 40 cm from the incisors (**Fig. 1A**). EUS demonstrated a hypoechoic mass with multiple cystic structures (**Fig. 1B**). The tumor had an unclear connection with the fourth layer (muscle layer) on EUS. Although EUS-guided fine-needle aspiration biopsy (EUS-FNA) was performed, a definitive diagnosis could not be established.

**Fig. 2:**
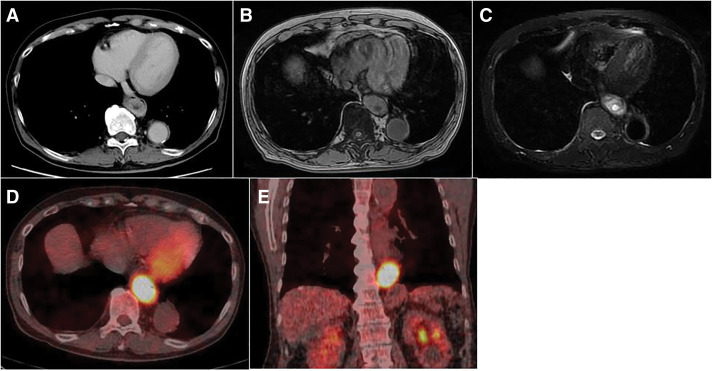
(**A**) Contrast-enhanced CT scan revealed a 60-mm SOL protruding into the lumen of the lower esophagus. The SOL exhibited peripheral enhancement with a central low-density area. (**B**, **C**) MRI revealed a SOL in the lower esophagus with high signal intensity on T2-weighted images and moderate signal intensity on T1-weighted images. The lesion contained cystic components exhibiting high T2 and low T1 signal intensities. (**D**, **E**) FDG-PET/CT revealed intense FDG uptake in the early phase, which further increased in the delayed phase (SUVmax: 11 → 18). SOL, space-occupying lesion; SUVmax, maximum standardized uptake value

Contrast-enhanced imaging revealed a 60-mm SOL protruding into the lumen of the lower esophagus. The SOL exhibited peripheral enhancement with a central low-density area (**Fig. 2A**). No enlargement of the adjacent or regional lymph nodes was identified. MRI revealed a SOL in the lower esophagus with high signal intensity on T2-weighted images and moderate signal intensity on T1-weighted images. The lesion contained cystic components exhibiting high T2 and low T1 signal intensities (**Fig. 2B** and **2C**). FDG-PET/CT (**Fig. 2D** and **2E**) revealed intense FDG uptake in the early phase, which further increased in the delayed phase (maximum standardized uptake value [SUVmax]: 11 → 18). No abnormal FDG accumulation suggestive of distant metastasis or lymph node involvement was identified. Given the preoperative diagnosis of a submucosal tumor in the lower thoracic esophagus, the presence of symptoms, the large size of the tumor, and the inability to exclude malignancy—particularly the possibility of a high-risk GIST with internal necrosis—surgical intervention was deemed necessary.

The surgery was performed via a laparoscopic approach. First, the abdominal esophagus was dissected, and the tumor was identified by intraoperative endoscopy. The esophagus was then transected proximal to the tumor by a linear stapler. A gastric conduit was constructed extracorporeally, and the overlap technique was used to perform an esophagogastric anastomosis. The operative time was 462 min, and the estimated blood loss was 80 mL. On POD 3, an anastomotic leak was detected, necessitating immediate reoperation. The leak was confirmed to have closed by POD 26 after the second surgery (day 29 after the initial surgery). The patient was discharged on POD 48 following the second surgery (day 51 after the initial surgery). During the first 3 postoperative years, he underwent annual surveillance with upper gastrointestinal endoscopy and thoracoabdominal contrast-enhanced CT. No evidence of recurrence or new lesion formation was detected. Given the benign nature of the lesion and the absence of clinical or radiological abnormalities during this period, routine surveillance was discontinued thereafter. The patient has remained recurrence-free for 7 years following surgical resection.

**Fig. 3:**
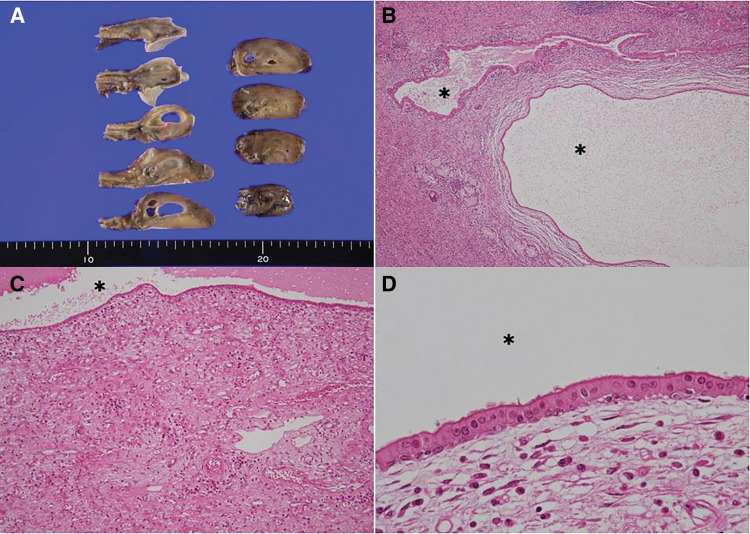
(**A**) Macroscopic examination of the tumor’s cut surface reveals multiple cystic structures of varying sizes within the lesion. (**B**, **C**) Histological analysis shows that the cysts are surrounded by inflammatory cell infiltration and granulation tissue. (**D**) The cyst walls are lined with columnar epithelium, with no evidence of malignancy observed (H&E staining). The asterisks indicate the cystic lumens. H&E, hematoxylin and eosin

Macroscopic examination of the cut surface of the resected specimen revealed cystic structures of varying sizes within the lesion (**Fig. 3A**). Histopathological examination (**Fig. 3B**–**3D**) revealed multiple cysts of varying sizes within the lamina propria. These cysts were surrounded by inflammatory cells and granulation tissue (**Fig. 3B** and **3C**), and were lined with columnar epithelium (**Fig. 3D**). Immunohistochemical staining was performed for CD34, c-kit, desmin, α-smooth muscle actin (SMA), and S-100, all of which were negative. These findings excluded the possibility of mesenchymal tumors such as GIST, leiomyoma, or neural-derived neoplasms. Based on the histopathological and immunohistochemical features, the resected specimen was diagnosed as an esophageal retention cyst.

## DISCUSSION

Esophageal retention cysts are rare, acquired cystic lesions characterized by dilation of the submucosal glands.^1)^ They are most commonly found in the distal esophagus but can develop throughout the esophageal tract. Endoscopically, they typically appear as polypoid or nodular lesions and may sometimes be misinterpreted as neoplastic growths.^2,3)^ While many cases remain asymptomatic, larger cysts can cause dysphagia, chest pain, or a sensation of a foreign body in the esophagus.

The pathogenesis of esophageal retention cysts is thought to involve ductal obstruction of the submucosal glands, resulting in the accumulation of glandular secretions and subsequent cyst formation. Esophagogastric junction outflow obstruction has been proposed as a contributing factor, as esophageal stasis may facilitate cyst development.^1)^ Histopathologically, these cysts are lined with cuboidal or columnar epithelium and may, in some cases, exhibit chondromatous metaplasia.^1)^ The differential diagnosis includes other benign esophageal cystic lesions, such as congenital duplication cysts, as well as malignant conditions.^9)^

PET/CT has been widely used for the diagnosis and staging of esophageal cancer, as it effectively detects primary tumors and distant metastases.^10)^ However, its utility for distinguishing benign from malignant lesions remains limited. Increased FDG uptake has been reported for various benign conditions that can mimic malignancies, including esophagitis, leiomyoma, schwannomas, and fibrovascular polyps,^5–8)^ and these findings are further detailed in **Table 1**, which summarizes previously reported benign esophageal lesions demonstrating FDG uptake on PET imaging. Notably, while the SUVmax values in earlier reports ranged from 3.6 to 12.3, the present case exhibited an exceptionally high SUVmax of 18, despite the lesion’s benign nature. This level of uptake exceeds that of other submucosal tumors, such as leiomyoma and schwannoma, and even surpasses the uptake observed in inflammatory esophagitis. These findings highlight the potential for certain benign esophageal lesions, particularly those with associated inflammation or cystic degeneration, to mimic malignancy on FDG-PET imaging, thereby complicating the diagnostic process.

**Table 1:** Reported cases of benign esophageal lesions with FDG uptake on PET/CT

Year	Author	Benign esophageal lesion	Clinical context	Size (maximum diameter)	SUVmax
2016	Jo et al.^5)^	Esophagitis	Reflex esophagitis (Los Angeles Classification grades C and D)	—	8.01 ± 0.38
2009	Miyoshi et al.^6)^	Leiomyoma	Submucosal tumor with FDG uptake	40 mm	4.7
2019	An et al.^7)^	Schwannoma	Submucosal tumor with FDG uptake	35 mm	12.3
2021	Nie et al.^8)^	Fibrovascular polyp	Submucosal rod-shaped lesion	150 mm	3.6
2025	Present case	Retention cyst	Submucosal tumor with cystic features	60 mm	11 → 18

FDG, fluorodeoxyglucose; SUVmax, maximum standardized uptake value

To further contextualize the mechanisms underlying FDG uptake in benign cystic lesions, similar findings have been reported in other organs. In a case of a splenic epidermoid cyst with hemorrhage, strong FDG uptake was observed in the cyst wall and was attributed to the presence of abundant inflammatory cells within the granulation tissue.^11)^ Similarly, foreign body granulomas caused by retained textiles often exhibit intense FDG uptake with a ring-shaped pattern, corresponding to a cellular foreign body reaction.^12)^ Additionally, in a case of a mucinous cystic neoplasm of the pancreas, inhomogeneous FDG accumulation in the cyst wall was associated with an ovarian-like stroma containing macrophages and fibrosis, rather than the epithelium itself.^13)^

The intense FDG uptake observed in our patient is particularly noteworthy, as most esophageal retention cysts are considered benign and typically exhibit minimal metabolic activity on PET/CT. Previous studies have reported that increased FDG uptake can occur in benign esophageal conditions, including esophagitis, granulomas, and certain benign tumors, occasionally mimicking malignancies.^5–8)^ The mechanism underlying FDG accumulation in esophageal retention cysts remains unclear; however, several explanations have been proposed.

One possible mechanism is chronic irritation and inflammation within the cyst, leading to increased metabolic activity. Esophageal cysts may be subjected to recurrent minor trauma from peristalsis or prolonged contact with luminal contents, which leads to local inflammation. Inflammatory cells, including activated macrophages and fibroblasts, have been shown to exhibit increased glucose metabolism, which may contribute to the observed FDG uptake.^14)^

In addition to these inflammatory processes, mechanical obstruction and esophageal stasis may also play a contributory role in the pathogenesis of esophageal retention cysts. Prior research has suggested an association between esophageal retention cysts and obstruction of the esophagogastric junction outflow.^1)^ Prolonged esophageal stasis could create a microenvironment that promotes cyst formation and secondary inflammation, further increasing metabolic activity. In our patient, histological examination of the resected specimen revealed infiltrating inflammatory cells and the presence of granulation tissue surrounding the cyst. These findings suggest that chronic irritation and inflammation within the cyst may have led to increased metabolic activity, thereby contributing to the observed FDG uptake.

For asymptomatic cases, conservative follow-up is generally recommended. However, in symptomatic cases, endoscopic resection is a viable and minimally invasive treatment option.^1)^ In general, the indication for endoscopic treatment of submucosal tumors is carefully determined based on multiple factors, including tumor size (typically ≤30 mm), the layer of origin, and the likelihood of benign or malignant pathology.^15,16)^ Surgical intervention is rarely required but may be considered for select cases. In this case, the patient presented with a symptomatic 60-mm tumor in the lower esophagus. EUS demonstrated an unclear relationship between the tumor and the fourth layer (muscularis propria), and a definitive diagnosis could not be established by biopsy, raising concerns about the risk of incomplete resection if endoscopic treatment were attempted. Furthermore, PET/CT revealed intense FDG uptake, raising a suspicion of malignancy, which led to the decision to perform a surgical resection as the most appropriate and definitive therapeutic approach. To the best of our knowledge, there have been no reported cases of FDG accumulation in esophageal retention cysts. Additional case studies are needed to increase our understanding of this phenomenon.

## CONCLUSIONS

In conclusion, this case highlights the diagnostic challenge of esophageal retention cysts presenting with elevated uptake of FDG. While PET/CT is a valuable tool in oncological imaging, clinicians should remain cautious when interpreting intense FDG uptake, as it does not necessarily indicate malignancy. Further research is needed to clarify the mechanisms underlying FDG accumulation in esophageal retention cysts and to refine the diagnostic criteria that differentiate benign from malignant esophageal lesions.

## ACKNOWLEDGMENTS

The authors would like to thank all individuals and organizations who contributed to the preparation of this manuscript. They also thank JAM Post (https://www.jamp.com/) for providing English language editing.

## DECLARATIONS

### Funding

The authors declare that they received no financial support pertaining to this case report.

### Authors’ contributions

BA acquired the data and drafted the manuscript.

HY, YO, and TT performed the surgery.

All other authors contributed to data collection and critically reviewed the manuscript.

All authors approved the final version of the manuscript and agreed to be accountable for all aspects of the work in ensuring that questions related to the accuracy or integrity of any part of the work are appropriately investigated and resolved.

### Availability of data and materials

Not applicable.

### Ethics approval and consent to participate

This case report was conducted in accordance with the ethical standards of the Declaration of Helsinki. Ethical approval was not required for this type of study in accordance with the institutional guidelines. Informed consent to participate in this study was obtained from the patient.

### Consent for publication

Written informed consent for publication of this case report was obtained from the patient.

### Competing interests

The authors declare that they have no competing interests.
